# Host and bacterial proteases influence biofilm formation and virulence in a murine model of enterococcal catheter-associated urinary tract infection

**DOI:** 10.1038/s41522-017-0036-z

**Published:** 2017-11-06

**Authors:** Wei Xu, Ana L. Flores-Mireles, Zachary T. Cusumano, Enzo Takagi, Scott J. Hultgren, Michael G. Caparon

**Affiliations:** 1 Department of Molecular Microbiology, Center for Women’s Infectious Disease Research, Washington University School of Medicine, Saint Louis, MO 63110-1093 USA; 2 Present Address: NextCure Inc., Beltsville, MD USA

## Abstract

*Enterococcus faecalis* is a leading causative agent of catheter-associated urinary tract infection (CAUTI), the most common hospital-acquired infection. Its ability to grow and form catheter biofilm is dependent upon host fibrinogen (Fg). Examined here are how bacterial and host proteases interact with Fg and contribute to virulence. Analysis of mutants affecting the two major secreted proteases of *E. faecalis* OG1RF (GelE, SprE) revealed that while the loss of either had no effect on virulence in a murine CAUTI model or for formation of Fg-dependent biofilm in urine, the loss of both resulted in CAUTI attenuation and defective biofilm formation. GelE^−^, but not SprE^−^ mutants, lost the ability to degrade Fg in medium, while paradoxically, both could degrade Fg in urine. The finding that SprE was activated independently of GelE in urine by a host trypsin-like protease resolved this paradox. Treatment of catheter-implanted mice with inhibitors of both host-derived and bacterial-derived proteases dramatically reduced catheter-induced inflammation, significantly inhibited dissemination from bladder to kidney and revealed an essential role for a host cysteine protease in promoting pathogenesis. These data show that both bacterial and host proteases contribute to CAUTI, that host proteases promote dissemination and suggest new strategies for therapeutic intervention.

## Urinary tract infections: targeting enzymes might help

Identifying bacterial and host enzymes that support biofilm formation may help prevent urinary tract infections caused by catheters. *Enterococcus faecalis* bacteria is a leading cause of catheter-associated urinary tract infections, the most common type of hospital-acquired infections. Michael Caparon and colleagues at Washington University School of Medicine in Missouri, USA, studied these infections in mice. They examined the effects of two protein-degrading enzymes, both from the bacterium and one can be activated by urine trypsin-like protease from the animals. Mutations that impaired either one of the enzymes had no effect on the infection, but when both the bacterial enzymes were impaired by mutation the formation of biofilms was significantly reduced. Treating the mice with chemicals that inhibited both bacterial and host enzymes dramatically reduced catheter-induced inflammation and related problems. This suggests drugs targeting these enzymes could be useful in clinical care.

## Introduction

Catheter-associated infections, particularly catheter-associated urinary tract infections (CAUTI), are the most common hospital acquired infections (HAI) worldwide and account for up to 40% of HAI in the USA.^1,2^ More than 560,000 patients develop CAUTI each year, which if untreated can lead to serious complications including bacteremia and death.^3–6^ Over the past few decades, *Enterococcus faecalis* has emerged as an important cause of CAUTI, whose treatment options are becoming increasingly limited due to its resistance to heat and aseptic solutions and its inherent and acquired resistances to multiple antibiotics, including vancomycin.^7,8^ Thus, understanding the molecular mechanisms of CAUTI pathogenesis is a critical need for the development of new antibiotic-sparing therapies.

One trait that has been established as important for the pathogenesis of enterococcal HAI and CAUTI is their ability to form biofilm on urinary catheters and other implantable devices.^9^ However, we have a relatively incomplete understanding of the genes and mechanisms used by enterococci to form biofilm in general and in the urinary tract environment in particular (for review, see ref. 10). One complication, is that in vitro assays for analysis of biofilm formation and growth are exquisitely sensitive to medium conditions and it is not always clear that conditions established for optimal formation of biofilm in vitro are representative of the in vivo environment encountered by enterococci during pathogenesis.^10^ For example, in the most widely used in vitro model (TSBG: trypicase soy broth +0.25% glucose), formation of biofilm structure is dependent on extracellular DNA (eDNA) released from enterococci by a fratricide mechanism that requires activation of the endogenous autolysin Atn in a minor population of cells within the biofilm.^11,12^ While deletion of the gene encoding Atn significantly reduces biofilm formation in the TSBG assay,^11–13^ these mutants are fully virulent and capable of forming catheter-associated biofilm in vivo in an optimized murine model of CAUTI,^14^ suggesting that the TSBG assay may not represent conditions encountered in CAUTI.

Insight into this discrepancy has come from analysis of the endocarditis and biofilm-associated (Ebp) pilus, an established virulence factor in the murine CAUTI model. Ebp has been shown to contribute to biofilm formation in the TSBG in vitro assay^15^ and to the formation of catheter biofilm in vivo in murine CAUTI.^16–18^ However, it has mechanistically different roles in each habitat. For the TSBG assay, Ebp promotes attachment of *E. faecalis* directly to several abiotic materials, including PVC, polystyrene, and silicone.^15,16^ In contrast, Ebp cannot promote attachment to abiotic substrates, including silicone catheter material, when exposed to urine, despite its role as a critical determinant for the attachment of *E. faecalis* to the catheter following its implantation into the murine bladder.^16^ This paradox was resolved when it was found that the catheter elicits an inflammatory response resulting in the release of the host protein fibrinogen (Fg) into the bladder lumen which coats the catheter.^16^ The pilus is tipped with the Fg-binding adhesin, EbpA, which binds to the Fg-coated catheter to initiate biofilm formation.^16^ Analysis of human urinary catheters supports a similar mechanism of Fg-mediated pathogenesis in human CAUTI^19^ and therapies that can block EbpA-Fg interaction are effective for both prophylaxis and treatment of CAUTI in the murine model.^16,20^ Taken together, these differences in the mechanism of Ebp-mediated biofilm among different habitats are mostly dependent on host and environmental factors and not the composition of the abiotic biofilm substratum.

These differences in host and environmental factors that affect biofilm formation in urine have been examined in greater detail, with the goal of developing an in vitro model that more accurately mimics CAUTI biofilm formation. While some *E. faecalis* strains have been reported to grow in urine,^21^ others grow very poorly and in all cases the efficiency of growth of any strain can be highly sensitive to the urine donor(s) (W. Xu, A. Flores-Mireles, S. Hultgren and M. Caparon, unpublished observations; M. S. Gilmore, personal communication). For example, the well-characterized *E. faecalis* strain OG1RF typically grows very poorly in urine in vitro, even though it is highly proficient in causing CAUTI in the murine model, with infection characterized by high overall bacterial burdens in the bladder and robust catheter-associated biofilm.^16^ However, if Fg is added to urine to mimic conditions encountered in the catheterized bladder, *E. faecalis* OG1RF grows several logs over the original inoculum, consumes Fg and forms biofilm on silicone catheters.^16^ Supplementation with BSA or casamino acids could also support growth, but only Fg was capable of promoting biofilm formation. Also, similar to biofilm formation in CAUTI, biofilm formed in Fg-urine is dependent on the Fg-binding activity of EbpA.^16^ These data show that a source of peptides can enhance growth in urine and that not only does Fg provide a critical substrate for attachment, that it can also serve as a key nutrient to support the growth of *E. faecalis* biofilm in the catheterized bladder.

The molecular details of how *E. faecalis* processes Fg and the biochemical pathways by which Fg supports growth are unknown. A complication is that Fg is a large glycoprotein (340 kDa), composed of three pairs of non-identical polypeptide chains: Aα-chain (610 amino acids, ~67 kDa), Bβ-chain (461 amino acids, ~55 kDa) and γ-chain (411 amino acids, ~48 kDa).^22^ For catabolism, bacterial generally lack the capacity to transport large polypeptides across their cellular membranes. Instead, they rely on secreted proteases to process large polypeptides into short peptides in the extracellular space that can then be transported into their cytoplasmic compartments by dedicated oligopeptide permeases.^23^ This suggests that the ability of *E. faecalis* to consume Fg as a growth substrate will be dependent on secreted proteases.

In this work, we examined the role of secreted proteases in the ability of *E. faecalis* to grow and form biofilm in an optimized model of CAUTI biofilm in vitro and for biofilm formation and pathogenesis in a murine model of CAUTI. Through analysis of mutant enterococcal strains defective in expression of several secreted proteases and through chemical inhibition of proteases we show that both bacterial and host proteases contribute to the ability of enterococci to utilize Fg during CAUTI and that host proteases play a major role in promoting inflammation and dissemination. Together, these data suggest that targeted inhibition of proteases can be developed into new antibiotic-sparing therapies for treatment of CAUTI.

## Results

### Enterococcal secreted proteases are required for optimum virulence in a murine model of CAUTI

**Fig. 1:**
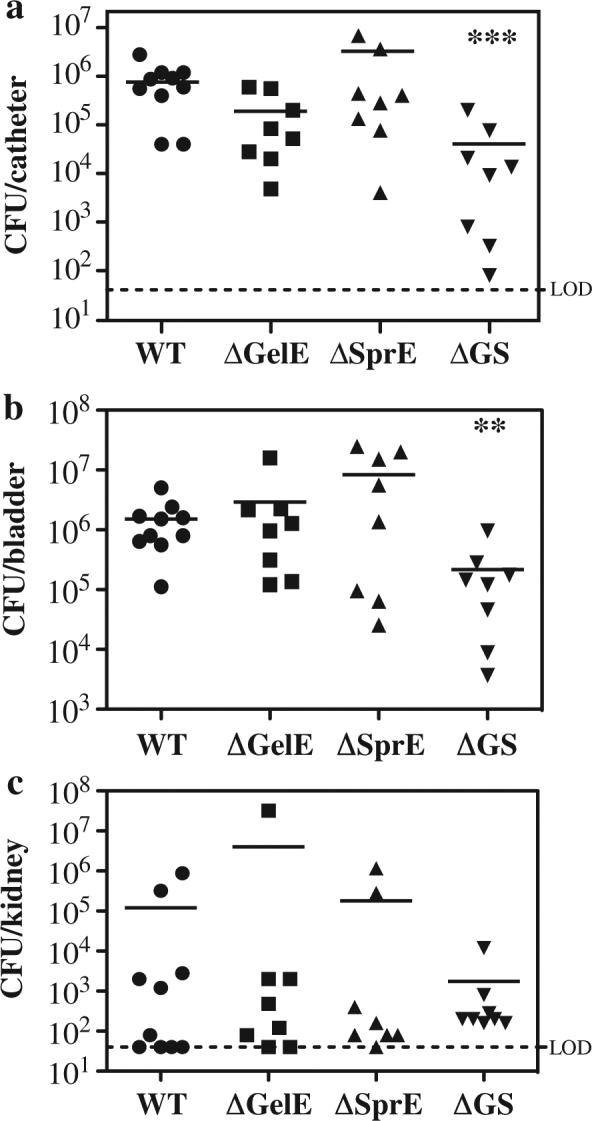
GelE and SprE are functionally redundant in CAUTI. Catheter-implanted mice were infected with ~2 × 10^7^ CFU of the indicated strains (see Table S1). Following 24 hrs of infection, total numbers of CFU recovered were determined for **a** Catheter, **b** Bladder and **c** Kidney. Each symbol represents an individual mouse and symbols touching the dashed lines indicate values below the limit of detection (LOD, 40 CFU). Data shown are pooled from two independent experiments. For each strain, the mean value is shown as the horizontal line. *p < 0.05, **p < 0.005, ***p < 0.001

Most strains of *E. faecalis*, including the well-characterized strain OG1RF and the prototype vancomycin-resistant (VRE) strain V583, express two secreted proteases, the metalloproteinase gelatinase (GelE) and a serine protease (SprE).^24^ The genes for both are co-transcribed from the same operon whose expression is under the control of the Fsr-quorum sensing system.^25,26^ To construct mutants deficient for expression of a single protease, in-frame deletion mutants were constructed in OG1RF that individually lacked *gelE* (ΔGelE) or *sprE* (ΔSprE), as well as a mutant that lacked expression of both proteases (ΔGS). All mutants had growth rates identical to the WT strain when examined in a standard rich medium (BHI, data not shown). The virulence of this panel of strains was then compared in our well-established murine model of CAUTI, which involves transurethral injection of bacteria into mice whose bladders have been implanted with a segment of silicone catheter.^14^ Virulence is quantitated by determination of the number of CFUs recovered from catheters, bladders and kidneys following 24 h of infection.^14^ This analysis revealed that the loss of an individual protease did not significantly alter colonization as compared to wild type (WT) with regards to CFUs recovered from the catheter (Fig. 1a), bladder (Fig. 1b) or kidney (Fig. 1c). In contrast, the mutant that lacked both proteases was significantly attenuated, with the number of CFUs recovered reduced by more than 1 log in all three compartments (ΔGS, Figs. 1a–c). Taken together, these data indicate that protease expression is required for optimum virulence in CAUTI, but that GelE and SprE are functionally redundant.

### Expression of both proteases is upregulated in human urine

**Fig. 2:**
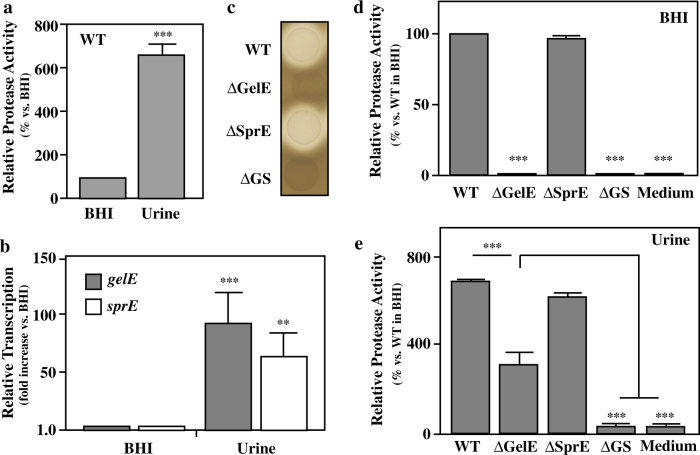
∆GelE has protease activity in urine, but not BHI. Expression of protease activity against a FITC-casein substrate **a** and relative transcription of the genes encoding the *E. faecalis* secreted proteases from the WT strain, as determined by real time RT-PCR **b** were assessed following 24 h of culture in the indicated medium. Protease activity of various strains (see Table S1) on protease indicator plates **c** and in supernatants from strains grown in BHI **d** or urine **e** following overnight culture. Values are normalized vs. culture density (OD_600_) and are compared to BHI **a**, **b** or compared to uninoculated media (Medium; **d**, **e**). On indicator plates, protease activity is apparent as a zone of clearing around the bacterial growth. Data are derived from a minimum of three independent experiments are shown as the mean ± SEM. For the indicated pair-wise comparisons, ***p < 0.001

To date, SprE has not been associated with virulence in other models of enterococcal infection. To gain insight into how SprE can promote virulence in CAUTI, the relative expression of each protease was examined following growth in BHI or in human urine. The expression of each protease was then quantitated by determination of protease activity in cell-free supernatants and by real time RT-PCR. Using a standard FITC-casein substrate, analysis of proteolytic activity revealed that overall caseinolytic activity was stimulated ~7 fold following growth in human urine vs. growth in BHI (Fig. 2a). Associated with this, expression of *sprE* and *gelE* was 50–100 fold higher in urine than in BHI (Fig. 2b). Expression of *fsrA* and *fsrB* was also ~100-fold elevated in urine and no protease activity was detected in a mutant with an in-frame deletion in *fsrB* during growth in urine (data not shown). These data show that the urinary tract environment is optimal for expression of both proteases and that their elevated expression in urine is dependent on the Fsr quorum-sensing system.

### SprE has proteolytic activity in urine but not in rich medium

Expression of GelE, but not SprE, correlated with protease activity on protease indicator plates, as ΔSprE (which expresses GelE) had caseinolytic activity equivalent to WT, while the mutants that lacked GelE (ΔGelE, ΔGS) lost all detectable activity (Fig. 2c). A similar pattern was observed following growth in BHI medium, as only cell-free supernatants from GelE^−^expressing stains (WT, ΔSprE) had activity against a FITC-casein substrate, while the mutant that expressed only SprE lacked all activity (ΔGelE, Fig. 2d). However, when grown in urine, both single mutants (ΔGelE, ΔSprE) had detectable protease activity. Activity was reduced as compared to WT (Fig. 2d) and in contrast to BHI medium, the mutant that only expressed SprE had detectable activity that was equivalent to ~40% of WT (ΔGelE, Fig. 2e). This activity was due to SprE, as deletion of the genes for both proteases reduced activity to that of urine alone (ΔGS, Fig. 2e). This latter activity was at a low, but detectable level (~3% of WT) suggesting urine has a low residual protease activity (Medium, Fig. 2e). As expected, expression of deleted genes in each respective mutant complemented mutant phenotypes in both BHI and urine (Fig. S1). These data show that unlike standard culture conditions, SprE contributes to proteolytic activity under conditions more representative of the urinary tract (UT) environment.

### In urine SprE can be activated by a GelE^−^ independent mechanism

**Fig. 3:**
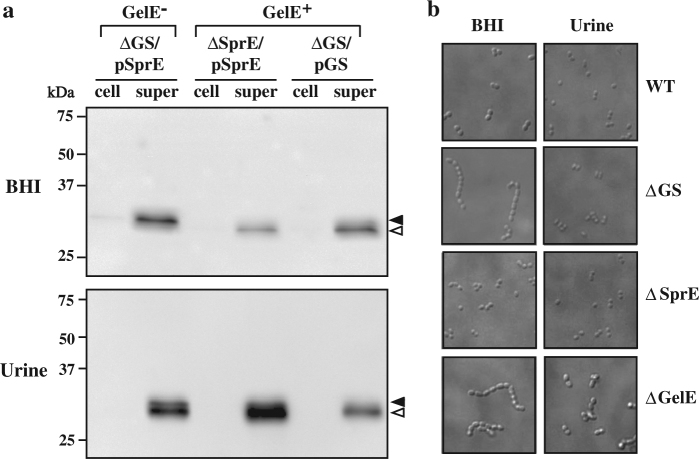
GelE-independent activation of SprE in urine. **a** GelE^−^ and GelE^+^ strains expressing SprE were constructed by complementing the indicated mutants with a plasmid expressing HA-tagged SprE (pSprE) or GelE and HA-tagged SprE (pGS). Supernatants (super) and cell lysates (cell) were then prepared following overnight culture in the indicated medium and subjected to a Western blot analysis to detect HA-tagged SprE. Arrows at the right indicate pro-SprE (closed) and mature SprE (open). Migration of several molecular weight standards are shown on the left. All blots are derived from the same experiment and processed in parallel. **b** Morphology of WT and ΔGS strains following overnight growth in the indicated medium was determined by phase contrast microscopy following fixation

Under standard growth conditions in BHI, the processing of the SprE zymogen to the active enzyme requires the protease activity of GelE.^27^ The observation above shows that SprE is active in urine in the absence of GelE, suggesting an alternative activation mechanism in this environment. To test this hypothesis, an epitope-tagged version of SprE was constructed and expressed from a plasmid in various mutants to derive GelE^+^ and GelE^−^ backgrounds. The processing of SprE was then monitored in immunoblots. Tagged SprE retained proteolytic activity (Fig. S1) and processing correlated with the appearance of a SprE polypeptide that was approx. four KiloDalton smaller that inactive SprE in immunoblots (Fig. S2). Following culture in BHI, the smaller active-associated form of SprE was only observed in culture supernatants from GelE^+^ strains and was not observed in GelE^−^ backgrounds (Fig. 3a). In contrast, following growth in urine, the smaller SprE band was now present in both GelE^+^ and GelE^−^ backgrounds (Fig. 3a). The loss of GelE activity has been associated with increased chain lengths, possibly through activation of a muramidase.^28^ Consistent with this, the loss of GelE, but not SprE, was associated with the formation of long multi-cellular chains of 10–15 cells following culture in BHI (Fig. 3b). The loss of both GelE and SprE lead to longer chains during growth in urine, although not to the same extend as in BHI medium, likely because of more limited growth (Fig. 3b). These observations suggest that while maturation of active SprE in BHI medium requires GelE, that the SprE zymogen can be activated independently from GelE in urine.

### Enterococcal secreted proteases are required for processing Fg

**Fig. 4:**
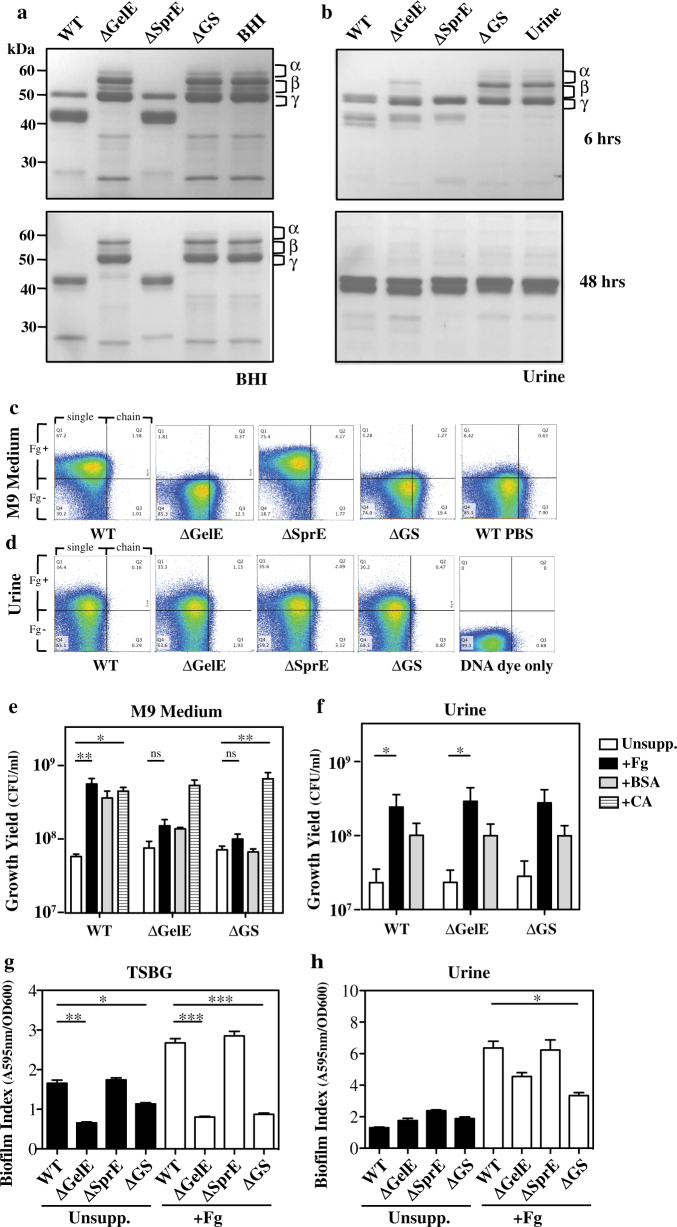
In urine GelE and SprE are functionally redundant for processing Fg and can enhance Fg-dependent biofilm formation. The ability of supernatants derived from the indicated strains (see Table S1) to process Fg is shown following overnight culture in BHI **a** or urine **b**. Cell-free supernatants were prepared, Fg was added and after an additional 6 or 48 h of incubation, the resulting cleavage patterns were resolved by SDS-PAGE and staining with Coomasie Brilliant blue for comparison to Fg incubated in uninoculated BHI (BHI) or urine (Urine). Migration of the Aα, Bβ, and γ chains of Fg and of several molecular weight standards are shown the right and left, respectively. All blots are derived from the same experiment and processed in parallel. The ability of alexa-fluor Fg to associate with the cell surface of the indicated strains following growth in optimized M9 medium **c** or urine **d** was analyzed by FACS. Enterococcal cells were counter stained with SYTO59. A PBS-washed WT and a SYTO59-only WT were used as control. Numbers in each panel indicates the percentage of the total number of cells contained within the indicated quadrant. The ability of the supplements indicated at the right to enhance growth in optimized M9 medium **e** or urine **f** was assessed by growth following overnight incubation by determination of CFUs. The ability of secreted proteases to enhance biofilm formation in the absence (unsupp.) or presence of Fg (+Fg) in TSBG **g** or urine **h** is shown. Biofilm formation by the indicated strains was analyzed following 48 h of incubation in 96-well plates by absorbance (A595) following staining with Crystal Violet. Biofilm was normalized with growth density measured at OD600. Data shown are from a minimum of three independent experiments and are shown as the mean ± SEM. For the indicated pair-wise comparisons, *p < 0.05, **p < 0.005, ***p < 0.001, *ns* not significant

Since Fg can promote the growth of *E. faecalis* OG1RF in urine in vitro, we examined whether the enterococcal-secreted proteases had the ability to cleave Fg. When examined following growth in BHI, supernatants from the WT strain cleaved Fg, preferentially making an initial cleavage in the Fg Aα, followed by cleavage in the Bβ and γ chains (Fig. 4a). Similar to caseinolytic activity, those mutants that lacked GelE lost the ability to process Fg into smaller peptides following growth in BHI (∆GelE, ∆GS; Fig. 4a), while processing was indistinguishable from WT in the mutant lacking expression of SprE (∆SprE, Fig. 4a). In contrast, when this same panel of mutants was grown in urine, the mutant that lacked both proteases could not cleave Fg, even after 6 h of incubation (∆GS, Fig. 4b), while the mutant that only expressed SprE was able to cleave Fg, although not as efficiently as WT (∆GelE, Fig. 4b). Upon extended incubation (48 h), no additional cleavage of Fg was observed in supernatants from BHI grown cultures or from incubation of Fg in uninoculated BHI (Fig. 4a). However, at this time point, Fg was cleaved upon exposure to normal urine alone, suggesting normal urine has protease activity against Fg (Fig. 4b). To gain insight into this protease activity, urines were supplemented with various inhibitors of the major classes of proteases. Only inhibitors of serine proteases, including trypsin inhibitor, could inhibit Fg cleavage (Table S3). Taken together, these data show that: i) the enterococcal secreted proteases are required for processing Fg; ii) while GelE is the dominant protease responsible for processing in a standard medium, both GelE and SprE contribute to processing in conditions more representative of the bladder ecology and; iii) there is a low level of a trypsin-like protease activity present in normal urine.

### Enterococcal proteases promote cellular association of Fg cleavage products

To examine the fate of the cleavage products of Fg, we developed an assay to detect the association of Fg with enterococcal cells. Since pigments in BHI were autofluorescent, this assay employed an optimized M9 minimal salt-based medium (“optimized M9,” see Materials and Methods). In this assay, fluorescently-labeled Fg (Fg-alexa fluor 488 conjugate) was added to optimized M9 or urine, which were then inoculated with various *E. faecalis* strains and cultured overnight. Aliquots were removed and the cellular association with Fg was assessed by flow cytometry. This assay revealed that following incubation in optimized M9, the WT strain demonstrated an ability to associate with the labeled Fg (WT, Fig. 4c), although this ability was lost if cells were washed in PBS prior to the addition of Fg (WT PBS, Fig. 4c). The ability of the Fg to associate with cells was dependent on the expression of GelE, as mutants that lacked GelE activity (∆GelE, ∆GS) had an approximately 10-fold lower ability to associate with Fg, and the mutant that only expressed SprE lacked the ability to associate with Fg (∆GelE, Fig. 4c). In contrast, following growth in urine, mutants that expressed either GelE or SprE demonstrated an ability to associate with Fg, as compared to the mutant that lacked both proteases (ΔGS, Fig. 4d). For cellular association, these data show that consistent with Fg processing behavior, GelE is required in artificial medium, but that GelE and SprE are functionally redundant under conditions more representative of the bladder environment.

### Host and enterococcal proteases promote Fg-dependent growth

The contribution of secreted proteases to the ability of Fg to enhance enterococcal growth was then examined. Consistent with prior results,^16^ *E. faecalis* OG1RF had a limited ability to grow in normal human urine, with a growth yield typically less than a 1 log increase over the initial inoculum (2 × 10^6^ cells/ml), which was reproduced with optimized M9 medium (Fig. 4e). In contrast, supplementing optimized M9 with Fg, bovine serum albumin (BSA) or casamino acids all enhanced growth yields by over 1 log (Fig. 4e). A similar result was observed for supplementation of urine with Fg or BSA (Fig. 4f). In optimized M9, the ability of Fg or BSA to enhance growth was dependent on GelE, as enhanced growth was not observed for mutants lacking GelE (∆GelE, ∆GS). Casamino acids enhanced growth (a source of amino acids and small peptides derived from hydrolysis of casein) independent of GelE (Fig. 4e), indicating that the proteolytic activity of GelE was required to process Fg and BSA into smaller peptides that could then be metabolized. In contrast, in urine, Fg or BSA could enhance growth of the mutant lacking either GelE or SprE (ΔGS, Fig. 4f), indicating that host proteolytic activity present in normal urine can sufficiently process Fg or BSA to promote enterococcal growth.

### Both SprE and GelE are required for optimal biofilm formation in Fg-urine

Next, we compared the panel of protease mutants for their ability to form biofilm in the standard TSBG and in the optimized Fg-urine assay. In TSBG, the WT formed a thick biofilm and the loss of GelE but not SprE, resulted in significantly less biofilm formation (compare ΔGelE, ΔGS to ΔSprE, Fig. 4g). In contrast, no strain was able to form appreciable levels of biofilm in the presence of urine (Fig. 4h). However, the addition of Fg enhanced biofilm formation of the WT strain in both TSBG (~2 fold, Fig. 4g) and in urine (~6 fold, Fig. 4h). In TSBG, the ability of Fg to enhance biofilm was dependent on GelE but not SprE, as mutants lacking the former demonstrated no increase in biofilm formation in the presence of Fg, while the mutant in the latter was indistinguishable from WT (ΔGelE and ΔGS vs. ΔSprE, Fig. 4g). Unlike the TSBG, the loss of either GelE or SprE did not alter biofilm formation in Fg-urine relative to WT (ΔGelE, ΔSprE, Fig. 4h). However, the loss of both resulted in significantly less biofilm formation in Fg-urine (ΔGS, Fig. 4h). The loss of both secreted proteases did not reduce biofilm formation in Fg-urine to the levels observed in the absence of Fg (ΔGS, Fig. 4h), consistent with the ability of normal urine to promote growth in Fg-urine as described above. Taken together, these data show that secreted proteases are important for the formation of Fg-enhanced biofilm and the combination of protease activity and Fg enhance biofilm formation to a much greater extend in urine than in TSBG. Furthermore, while GelE is the major protease required for Fg-enhanced biofilm formation in TSBG, host proteases may contribute to Fg-dependent biofilm formation in urine. However, the enterococcal-secreted proteases are required for optimal biofilm formation in Fg-urine, although unlike Fg-TSBG, GelE, and SprE are functionally redundant in the Fg-urine assay.

### Chemical inhibition of proteases improves outcome in murine CAUTI

**Fig. 5:**
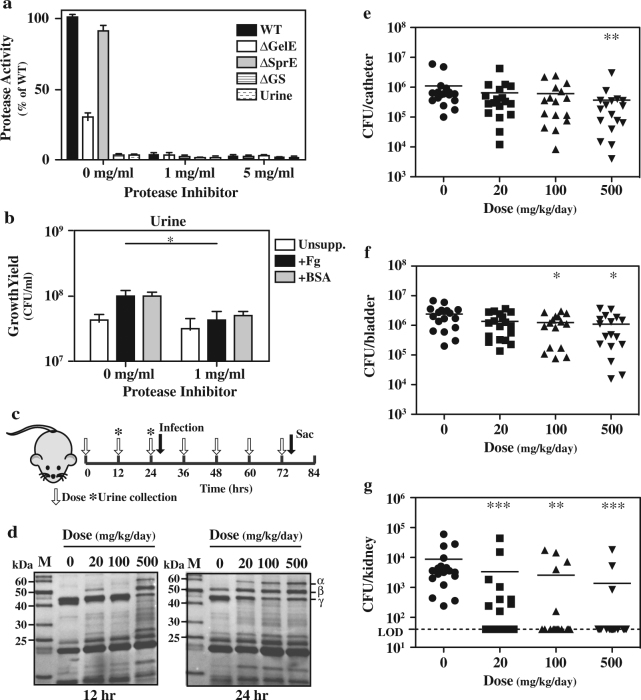
Protease inhibitor treatment reduced bacterial burden in CAUTI. **a** The ability of a protease inhibitor cocktail (cOmplete™ Mini) to inhibit *E. faecalis* protease activity was assessed following overnight growth of the indicated strains in urine. Protease activity in supernatants against a FITC-casein substrate was then determined. **b** The growth of the WT strain in urine unsupplemented (unsupp.) or supplemented as indicated, in the presence or absence of the inhibitor cocktail was assessed by determination of CFUs following overnight growth. **c** Experimental timeline for treatment of murine CAUTI. The inhibitor cocktail was administered by intraperitoneal injection (dose) in four groups of mice at 0, 20, 100, and 500 mg/kg. Infection: time of catheter implantation followed immediately by infection by the WT strain. Sac: time of mouse sacrifice. **d** Urines collected at the indicated time points were tested for inhibition of endogenous protease activity by the addition of Fg followed by SDS–PAGE as described above (Fig. 4). The migration of several molecular weight standards is shown (M) and that of the Fg chains shown on the left. All blots are derived from the same experiment and processed in parallel. Following sacrifice, bacterial burdens were determined in catheter **e**, bladder **f** and kidney **g**. Total CFU recovered is shown. Each symbol represents an individual mouse. Symbols touching the dashed line indicate that recovery was below the limit of detection (LOD, 40 CFU). Infection data are pooled from two independent experiments. For each dose, the mean value is shown as the horizontal line. Other data are shown as the mean ± SEM derived from at least three independent experiments. *p < 0.05, **p < 0.005, ***p < 0.001

Since the data presented above show that secreted enterococcal proteases enhance CAUTI pathogenesis, we investigated whether treatment with several commonly used protease inhibitors could alter the course of pathogenesis in the murine CAUTI model. A commercially available protease inhibitor cocktail (cOmplete™, Mini, Roche) was effective at inhibiting caseinolytic activity from both host and bacterial proteases during growth in vitro in urine (Fig. 5a) and for inhibiting the ability of Fg- or BSA to enhance enterococcal growth in urine (Fig. 5b). Groups of C57BL/6NCI mice received a range of doses of the cocktail (0, 20, 100, and 500 mg/kg/day) delivered intraperitoneally according the schedule described in Fig. 5c. Urines were collected following 12 and 24 h (Fig. 5c) and tested for inhibitory activity by adding Fg, incubating the mixture overnight and assessing the cleavage of Fg by SDS-PAGE. This analysis revealed that the highest dose (500 mg/kg/day) had a detectable ability to block Fg degradation by host proteases after one injection (12 h, Fig. 5d), while inhibitory activity was detectable by the lowest dose tested after two injections (20 mg/kg/day, Fig. 5d), indicating the presence of therapeutic concentrations of the inhibitors. Following the second dose at 24 h, mice were implanted with catheters and infected and then received additional doses of the cocktail every 12 h over the course of a 72 h experiment (Fig. 5c). At this point, bacterial burdens on catheter, bladder and kidney were evaluated by determination of recoverable CFU. This analysis revealed that burdens were; i) significantly reduced as compared to vehicle (PBS) on catheters at the highest dose (500 mg/kg/day, Fig. 5e); ii) significantly reduced at the two highest doses in bladder (100, 500 mg/kg/day; Fig. 5f) and; iii) reduced at all doses in kidney (20, 100, 500 mg/kg/day; Fig. 5g). Most strikingly, protease inhibitor treatment not only reduced bacterial burdens in kidneys, but significantly blocked dissemination to kidneys, as defined by reducing recoverable CFU to below the limit of detection (LOD, Fig. 5g).

### Inhibition of host cysteine protease activity alters pathogenesis in CAUTI

**Fig. 6:**
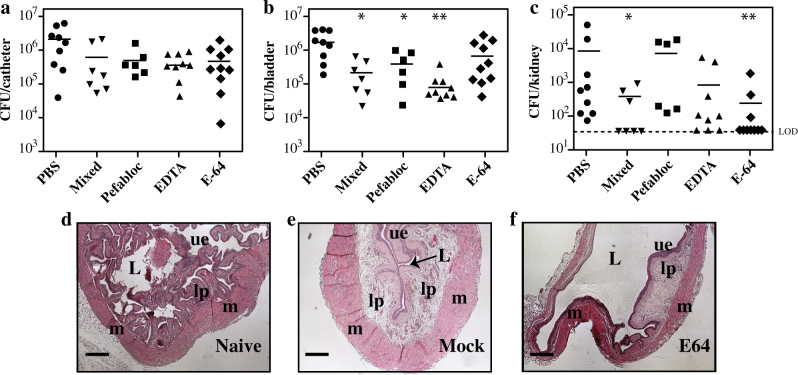
A cysteine protease inhibitor reduces dissemination. Groups of mice were treated with the protease inhibitors indicated or mock-treated (PBS), infected and analyzed as described in Fig. 5 for determination of bacterial burdens in catheter **a**, bladder **b** and kidney **c**. Inhibitors were used at the following concentrations: Pefabloc, 50 mg/kg/day; EDTA, 30 mg/kg/day; E64, 2 mg/kg/day; Mixed: Pefabloc + EDTA + E64 (50 mg/kg/day, 30 mg/kg/day, 2 mg/kg/day respectively). Histology of bladders in H&E-stained section is shown for mice **d** uninfected (naïve), **e** catheter-implanted and infected (mock) and **f** E64-treated, catheter-implanted and infected by the WT strain. Labels are: *L* lumen; *lp*, lamina propria, *ue* urothelium, *m* muscularis. Scale bars: 200 µm. *p < 0.05, **p < 0.005

Since the inhibitor cocktail targets multiple classes of proteases, we next examined the effect of inhibitors targeting specific classes of proteases. Well-characterized non-toxic inhibitors targeting serine proteases (Pefabloc), metalloproteases (EDTA), cysteine proteases (E64), and a mixture (Pefaloc + EDTA +E64) were used to treat infected mice as described above (Fig. 5c). While the various treatments did not alter the numbers of recoverable CFUs on catheters as compared to mock (PBS) treated mice (Fig. 6a), bacterial burdens were significantly reduced in bladders by Pefabloc and EDTA (Fig. 6b). Most strikingly, bacterial burdens in kidney were significantly reduced by the combination of Pefabloc, EDTA, and E64 (“mixed”) and by E64 alone (Fig. 6c). This indicates that E64 was sufficient to decrease dissemination. To examine this latter effect in greater detail, the histology of E64-treated bladders was examined following 48 h of infection. While un-catheterized and uninfected bladders showed the characteristic folds of urothelium projecting into the lumen (Naïve, Fig. 6d), bladders that were implanted with catheters, but were not infected, were inflamed as previously described^7,14,16^ with an edematous lamina propria that has expanded to occlude much of the lumen (Mock, Fig. 6e). Numerous studies have shown that infection of catheter-implanted bladders by *E. faecalis* typically does not produce further alterations to bladder tissue architecture.^7,14,16,29^ However, infected catheter-implanted bladders treated with E64 have a markedly different appearance. These bladders appeared distended with markedly reduced edema of the lamina propria (Fig. 6f) and this was consistently observed for all bladders examined (*N* = 8, Fig. S3). Given that the genome of *E. faecalis* OG1RF does not encode any apparent cysteine protease that can be targeted by E64,^30^ this result suggests that inhibition of a host cysteine protease can influence the pathogenesis of CAUTI.

## Discussion

Using a combination of mutants and chemical inhibition, we show that both enterococcal and host proteases contribute to the pathogenesis of *E. faecalis* CAUTI. The enterococcal secreted proteases were required for growth and biofilm formation in Fg-supplemented urine, a condition more representative of the CAUTI habitat than traditional in vitro assay conditions. The enterococcal proteases were also required for biofilm formation and virulence in a murine model of CAUTI. Furthermore, a host cysteine protease promotes inflammation in the catheterized bladder that facilitates dissemination of the infection to the kidney. These data suggest that a strategy targeting inhibition of specific proteases can be developed to augment existing therapies against CAUTI and disseminated infection. This approach will likely prove efficacious against other pathogens, including *Staphylococcus aureus* and *Candida albicans*, that are also known to exploit Fg for CAUTI pathogenesis.^19^

This study contributes to our understanding of the important role of Fg in catheter-biofilm formation in promoting both enhanced growth in urine and better biofilm. Several unexpected results, including that: (i) GelE and SprE are functionally redundant for CAUTI pathogenesis; (ii) there is a GelE-independent pathway for activation of SprE in the urinary tract and; (iii) SprE can support CAUTI pathogenesis independent of GelE. Traditionally, GelE has been recognized as the secreted protease that plays a major role in the pathogenesis of multiple enterococcal diseases, including peritonitis and endocarditis.^28,31–33^ There is considerably less information on the possible contribution of SprE to pathogenesis. Its best-characterized role is in eDNA-dependent biofilm formation, where it can modulate the amount of cell lysis by modifying AltA autolysin against further proteolytic activation by GelE.^31^ However, consistent with the data presented here, a requirement for GelE to activate SprE under most in vitro conditions,^27^ means that the loss of GelE is epistatic on SprE activity. Thus, a GelE mutant will lack both GelE and SprE activities, making it difficult to parse out SprE’s specific contributions. A different situation was observed in urine, as a host protease could activate SprE independently of GelE. The fact that both proteases had activity against Fg can explain why they were functionally redundant in urine and that the loss of both was required for attenuation in CAUTI, consistent with our prior observation that disruption of GelE alone did not result in reduced virulence in CAUTI.^14^

The presence of a host protease with activity against Fg can also explain why the ΔGS mutant had enhanced growth in Fg-urine in vitro, despite its lack of both GelE and SprE. This observation is consistent with a prior study that reported a proteolytic activity in normal urine active against albumin.^34^ Consistent with this, a proteomic comparison of proteins associated with an implanted catheter between uninfected and infected mice, revealed that the precursor of the serine protease trypsin was associated with catheters recovered from uninfected mice (Table S4), suggesting that the SprE-activating activity in urine was possibly due to trypsin. Consistent with this, trypsin can activate SprE in vitro (Fig. S4). However, the situation is likely to be more complicated during CAUTI vs. normal urine, as: (i) the levels of the trypsin precursor were decreased below the limit of detection after 24 h of infection and; (ii) infection resulted in the accumulation of several trypsin/serine protease inhibitors on the catheter (Table S4). A possible source of these inhibitors may be the PMN’s recruited into the bladder during CAUTI.^7^ These infection-induced changes could affect the dynamics of host and bacterial protease interaction during CAUTI and may explain why host protease activity could activate SprE, but not support the pathogenesis of the ΔGS mutant during CAUTI. Further analysis will be required to understand how host and enterococcal proteases and their inhibitors interact during CAUTI.

The functional redundancy of GelE and SprE also raises some interesting questions about the role of the enterococcal-secreted proteases in CAUTI pathogenesis. For example, it is not clear why there is selection to maintain both proteases, when either one is sufficient to support CAUTI. A broad substrate specificity for GelE has been described, including degrading insulin β-chain, bradykinin, and the antimicrobial peptide LL-37.^35–37^ The substrates of SprE have not been as well defined, although the data here show that both SprE and GelE can cleave Fg. Similar to a cysteine protease (SpeB) in *Streptococcus pyogenes* and a metalloprotease (PrtV) in *Vibrio cholerae*,^38,39^ the present study shows that *E. faecalis* preferentially makes an initial cleavage in the Fg Aα-chain followed latter by cleavage in the Bβ and γ chains. However, at the earlier time points in urine, the cleavage patterns between WT, ΔGelE, and ΔSprE are not identical, suggesting that optimal processing of Fg requires a synergistic interaction of the two secreted proteases. In addition, the cleavage patterns of all strains are different between BHI and urine grown cultures, suggesting that a host protease also contributes to Fg processing in this environment.

Our studies using chemical inhibitors of protease activity implicated the contribution of a host cysteine protease to pathogenesis that functioned to promote dissemination of the infection to the kidney. This is consistent with reports that host proteases can profoundly influence pathogenesis, including both anti-inflammatory and pro-inflammatory effects.^40^ For example, GelE may have an anti-inflammatory activity by virtue of its ability to degrade the C3 component of complement.^41^ Excessive proteolytic activity can lead to maladaptive host responses and excess tissue inflammation and damage. Several classes of host cysteine proteases, including caspases, cathepsins and calpains, have well characterized regulatory roles in the pro-inflammatory response. A common theme involves the proteolytic activation of pro-inflammatory cytokines. For a few examples, danger signals that activate the inflammasome lead to the caspase-family dependent activation of IL-1β and other cytokines,^42^ while calpain-family proteases participate in the inflammasome-independent activation of IL-1α.^43^ Of interest, IL-1β and IL-1α can play distinct roles in inflammation, with the former more important for infection, while the latter is more important for sterile inflammation (for review, see ref. 44), similar to what may be induced during placement of a catheter. Prior results indicate that IL-1β is upregulated when catheter-implanted bladders are infected by *E. faecalis*,^14^ although the mechanisms responsible for catheter-induced inflammation in the absence of infection have not been as well characterized.^7^ However, our observation that dissemination and inflammation are reduced by inhibition of a host cysteine protease implicates inflammation in the control of dissemination and that this cysteine protease regulates this response. Optimization of protease-inhibitor therapy will require the identification of this regulatory cysteine protease so that it can be targeted by more specific inhibitors.

Protease inhibitor therapies have proven successful for treatment of a constantly expanding list of diverse infectious diseases. For example, a cysteine protease inhibitor (K11777) rescued mice from lethal infections by *Cryptosporidium parvum*, a protozoan parasite causing stunted infant growth and lethality in immunocompromised individuals.^45^ Nine protease inhibitors targeting pathogen proteases have been approved for clinical use against HIV^46^ and inhibitors of viral proteases have been successful in eradicating infection by Hepatitis C virus.^47^ Therapy directed against host proteases have also shown efficacy as a serine protease inhibitor (serpinb1) acted to restore tissue homeostasis by regulating the innate immune response to protect lungs from damage by infection with *Pseudomonas aeruginosa* in a murine model.^48^ Interestingly, mutants of *E. faecalis* lacking the intramembrane protease Eep also showed reduced kidney dissemination in the murine CAUTI model.^49^ This protease is involved in maturation of an enterococcal pheromone (cCF10) involved in cell-to-cell communication and DNA transfer and its activity is sensitive to chemical inhibition.^50^ This suggests that by expanding the repertory of targeted proteases from the secreted proteases to include various cell-associated proteases, the efficacy of protease inhibitor therapy in CAUTI can be improved.

Short-term urinary catheterization increases the risk of developing a UTI and other complications up to 80%, and prolonged catheterization can increase the risk to 100%.^51–53^ The commensal Gram-positive bacterium *E. faecalis* has a remarkable ability to adapt to the UTI environment by forming biofilm on catheters, which provides it with a conduit for ascension through the catheter lumen into the bladder.^7^ Biofilms can be particularly challenging from an infection control standpoint, as they are often refractory to antibiotics and the host immune system. In prior work we have shown that Fg plays a critical role in catheter biofilm formation by virtue of its ability to coat catheter surfaces.^19,20^ Since completely blocking Fg deposition on catheters is likely to prove challenging, we have instead focused on developing therapies to block enterococci from binding to Fg.^16,20^ In this study, we capitalized on the prior observation that Fg can enhance growth under conditions representative of CAUTI environment,^16^ and the observation made here that expression of the secreted proteases GelE and SprE are highly up-regulated upon exposure to Fg-urine to examine how Fg is processed by these proteases to enhance biofilm formation and growth. Taken together, these findings will guide the development of new therapies that will improve the efficacies of current treatments and provide a platform to study the role of the inflammatory response in CAUTI.

## Methods

### Bacterial strains, media, and growth conditions

Molecular cloning experiments utilized *Escherichia coli* TOP10 cultured in Luria–Bertani medium. Experiments involving *E. faecalis* used strain OG1RF and several mutant derivatives (see below and Table S1) that were regularly maintained on Brain Heart Infusion (BD, Franklin Lakes, NJ) (BHI) agar plates supplemented with 25 μg/ml of rifampin and 25 μg/ml of fusidic acid.^54,55^ Liquid media included BHI, Trypticase Soy Broth (BD) + 0.25% glucose (TSBG), human urine (see below), or optimized M9 medium (see below). Where indicated, liquid media were supplemented with Fg (Sigma-Aldrich, St. Louis, MO), BSA (Sigma-Aldrich), or Casamino Acids (BD) by the addition of lyophilized powder directly to media to achieve a final concentration of 1 mg/ml. Unless otherwise specified, cultures in liquid media were inoculated from a single colony and grown statically at 37 °C for 18 h. Bacterial titers were determined as previously described.^16^ Where appropriate, antibiotics were added at the following concentrations: *E. faecalis*: erythromycin, 30 μg/ml; kanamycin 500 μg/ml; and chloramphenicol 10 μg/ml. *E. coli*: erythromycin, 500 μg/ml; kanamycin, 50 μg/ml; and chloramphenicol 10 μg/ml.

### Construction of in-frame deletion mutants and complementation

The *gelE* deletion mutant described previously^12^ is referred to in this study as ∆GelE (Table S1). In-frame deletion mutations in other genes were generated by allelic replacement using a standard method.^18^ Briefly, the upstream and downstream sequences flanking the open reading frame of interest were amplified by PCR using the oligonucleotide primers listed in Table S2 and inserted into the allelic replacement vector pGCP213 through overlap extension PCR.^56^ The resulting plasmids were the introduced into the relevant OG1RF host and allelic replacement conducted as described.^18^ Where indicated, full length open reading frames of *gelE*, *sprE*, and *gelE-sprE* were fused with a C-terminal HA tag into the *E. coli*/*E. faecalis* shuttle vector pABG5 as previously described.^57^ These constructs (Table S1) were transformed into the corresponding protease deletion mutants for phenotypic characterization (see below). The fidelity of all constructs and mutant chromosomes was confirmed by DNA sequence analysis (Genewiz, South Plainfield, NJ) of PCR products generated using the relevant primers (Table S2).

### Collection of urine

To minimize influence of donor variability, human urine was collected and pooled from three healthy female donors between 20–35 years of age, who have no history of kidney disease, a current UTI, diabetes or were currently undergoing antibiotic treatment. Urine was sterilized by filtration through a 0.22 μm filter (EMD, Milipore) and adjusted to pH 6.0–6.5 prior to use. All participants have signed an informed consent and the forms are in compliance with local confidentiality laws. Where indicated, urine was mimicked using M9 medium (BD) supplemented with 0.04% (wt/vol) glucose, 9.3 mg/ml urea, 0.6 mg/ml creatinine, 1.29 mg/ml Na_3_C_6_H_5_O_7_, 2 mM MgSO_4_, 0.1 mM CaCl_2_ and 0.005–0.03% yeast extract,^58^ referred to as “optimized M9 medium.”

### Protease expression and activity

The ability of strains to express protease activity were assessed following growth on protease indicator plates^59,60^ and from the supernatants of liquid cultures using an FITC-casein substrate, normalized to culture density (OD_600_), was determined as described.^61^ The ability of the various strains to express HA-tagged protease polypeptides was conducted by subjecting culture supernatants that were clarified by centrifugation and filter-sterilized to a Western blot analysis using a mouse monoclonal anti-HA antibody (Sigma-Aldrich, H3663, dilution 1:2000) that was detected using a goat anti-mouse IgG conjugated with HRP (Sigma-Aldrich, A4416, 1:2000). Blots were developed and imaged using a ChemiDoc PM system (Bio-Rad), as previously described.^62^ For activity in whole cell lysates, cells from overnight culture were harvested by centrifugation, washed twice in PBS and resuspended in 1 × SDS-sample buffer. The mixture was then placed in boiling water bath for 5 min. immediately prior to analysis by SDS-PAGE as described above. For analysis of protease gene expression, RNA was isolated using Direct-zol^TM^ RNA miniprep kit (Zymo research) per the manufacture’s protocol. Reverse transcription, RT-PCR were performed using the primers listed in Table S2 as previously described.^63^ Relative transcript abundance was determined by the –ΔΔC_T_, method normalized to 16 s rRNA expression and relative to wild type as described.^63^ Unless otherwise indicated, activities were measured following overnight culture.

### Analysis of Fg processing and flow cytometry

For analysis of Fg cleavage, culture supernatants were collected following overnight culture and were clarified by centrifugation and sterilized by filtration. Bovine Fg (Sigma-Aldrich) was then added to a final concentration of 1 mg/ml and the mixture incubated at 37 °C for the time periods designated in the text. Samples were then mixed with an equal volume of 2 × SDS-sample buffer and subjected to analysis by SDS-PAGE. Digestion patterns were then assessed by staining with Coomassie Brilliant Blue R by standard methods. For analysis of Fg-cell interaction by flow cytometry, strains were cultured overnight in optimized M9 medium or urine, each supplemented with Alexa-Fluor 488 labeled human Fg (Thermo-Fisher Scientific, F13191) at a final concentration of 10 ng/ml. Bacterial samples were analyzed directly from culture. Bacterial cells were counterstained with SYTO^®^59 red fluorescent nucleic acid stain (Thermo-Fisher Scientific, S11341) (5 μM) 10 min. prior to flow cytometry and the amount of Fg associated with the cells was determined using a FACSCalibur flow cytometer (BD Biosciences) with analysis using FlowJo software (FlowJo, LLC).

### Biofilm formation

Routine quantitation of biofilm formation was conducted following overnight growth in the various media listed in the text in 96-well polystyrene plates by the crystal violet staining method as previously described.^16^

### Mouse catheter implantation, infection, and treatment

Mice used in this study were six-week-old female wild-type C57BL/6Ncr mice purchased from Charles Rivers Laboratories that were transurethrally implanted with a 5-mm length of platinum-cured silicone catheter as previously described.^14^ Animal chosen were randomized. Where indicated, mice were transurethrally infected immediately after implantation with a dose of ~2 × 10^7^ CFU in PBS in a total volume of 50 µl. Mock infected mice received 50 µl of PBS alone. For protease inhibitor therapy, tablets of the inhibitor (cOmplete™ Mini, catalog #11836153001, Roche) were dissolved in PBS according to the Manufacturer’s recommendations to achieve the concentration stated in the text. Mice received intraperitoneal injections of 100 ul of the inhibitor solution, PBS alone (mock) and were implanted and infected according to the schedule described in Fig. 5c. Urine was collected at the designated time points (Fig. 5c) for analysis of Fg cleavage as described above and bacterial titers in the catheter, kidney or bladder were determined as previously described.^7^ Other inhibitors are described in Fig. 6. Mice loss implanted catheters were excluded from data analysis.

### Histological analyses, microscopy and image processing

For histological analyses, bladders were fixed in 10% formalin for 24 h and dehydrated in 70% ethanol overnight at 4 °C. They were then embedded in paraffin, sectioned, and stained with Hematoxylin and Eosin (H&E) for light microscopy. Images were acquired using Leica DM1000 microscope equipped with an EC3 digital camera. The histological images presented were stitched together from multiple overlapping images using the Photomerge function of Adobe Illustrator CC (ver. 2017.1.0) or LAS EZ software (*Leica Microsystems* **)**. For publication, images were processed using Adobe Photoshop CC (ver. 2017.1.1) and prepared using Adobe Illustrator CC (ver. 2017.1.0).

### Statistical analyses

Data from multiple experiments were pooled. For experiments involving infection, each strain was tested in a group of 5 mice, with independent repetitions. The difference between WT and each mutant strain was tested for significance using a two-tailed Mann–Whitney *U* test. For other experiments, data are derived from at least 3 independent experiments, with values presented representing the mean ± SEM for each group with differences tested for significance using a paired *t*-test. Values below the limit of detection (LOD) for CFU assays were assigned the LOD value (40 CFU). All statistical tests were performed using GraphPad Prism software (ver. 5, GraphPad Software). For all statistical tests, the null hypothesis was rejected for *p* < 0.05. In Figures, *p* values for specific comparisons were indicated using the following symbols: <0.05 (*), <0.005 (**), and <0.001 (***).

### Study approval

Urine samples were collected as per the study approval from the Washington University School of Medicine Internal Review Board (approval ID #201207143). All procedures involving animals were reviewed and approved the Animal Studies Committee of the Washington University School of Medicine.

### Data availability

All data that support the findings of this study are available from the corresponding author upon reasonable request.

## Electronic supplementary material

Supplementary Materials
