# Supplementary material for: Host and bacterial proteases influence biofilm formation and virulence in a murine model of enterococcal catheter-associated urinary tract infection
Source: NPJ Biofilms Microbiomes. 2017 Nov 6;3:28. doi: 10.1038/s41522-017-0036-z (PMC5673934; doi:10.1038/s41522-017-0036-z)
Supplement: Supplementary file 1 — Supplementary Materials [file 41522_2017_36_MOESM1_ESM.pdf]

SUPPLEMENTARY MATERIALS, FIGURES AND TABLES

Supplementary Methods

Mass Spectrometry. Catheters were retrieved from bladders, and boiled in SDS-sample buffer to release proteins. Samples then sent to Proteomics and Mass Spectrometry Facility at the Danforth Plant Science Center (St. Louis, MO) for analysis. Proteins samples were digested with trypsin at 37°C overnight. The extracted peptides were dried down and each sample was resuspended in 100 µL 1% ACN/1% FA. 5 µL were injected and run on an LTQ-Orbitrap Velos Pro on a Dionex RSLCnano HPLC using a 1h gradient. All MS/MS samples were analyzed using Mascot (Matrix Science, London, UK; version 2.5.1.0). Mascot was set up to search the cRAP_20110301 and NCBInr database (selected for Mus musculus) assuming the digestion enzyme trypsin. Scaffold (version Scaffold_4.4.5 Proteome Software Inc., Portland, OR) was used to validate MS/MS based peptide and protein identifications. This material is based upon work supported by the National Science Foundation under Grant No.DBI-0922879 for acquisition of the LTQ-Velos Pro Orbitrap LC- MS/MS.

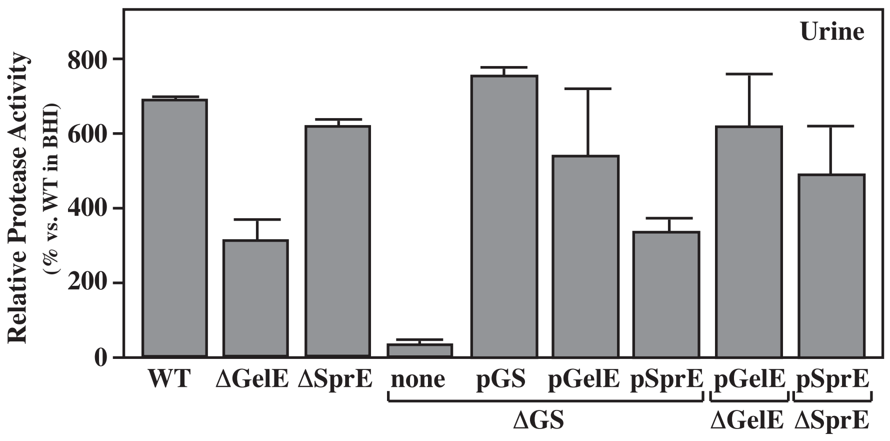

Figure S1. Complementation of protease mutations. Protease deletion mutants (ΔGelE, ΔSprE. ΔGS; see Table S1) were complemented with plasmids expressing GelE (pGelE), SprE (pSprE) and both GelE and SprE (pGS) or not complemented (none), as indicated. Following overnight culture in urine, protease activity in cell-free supernatants was quantitated using a FITC-casein substrate. Data are presented relative to WT grown in BHI medium. Shown is the mean ± SEM from a minimum of 3 independent experiments.

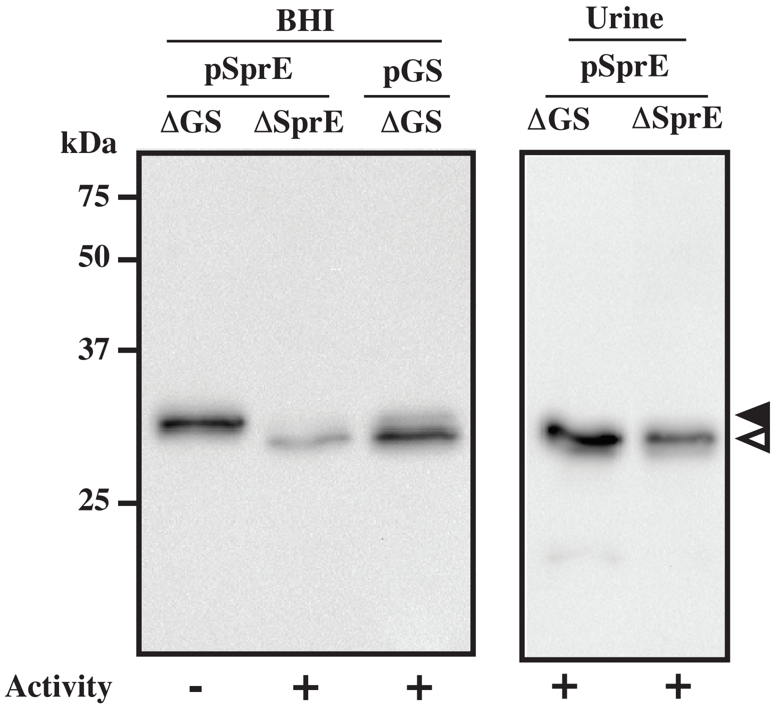

Figure S2. Inactive pro-SprE zymogen is processed by GelE. Protease deletion mutants were complemented with various plasmids to generate strains that express HA-tagged SprE in the absence of GelE activity (ΔGS/pSprE) or the presence of GelE activity ((ΔSprE/pSprE, ΔGS/pGS) (Table S1). Following overnight culture in BHI/urine, supernatants were subjected to 1) a Western blot analyses to detect HA-SprE shown at the top of the Figure and 2) protease activity assay against a FITC-labeled casein substrate, shown at the bottom of the Figure (+: positive protease activity; -: no protease activity). This assay reveals that the presence of GelE, correlates with the appearance of a 4kd smaller form of SprE. Note that although SprE has the capacity to cleave FITC-casein during culture in urine, the unprocessed SprE produced in the absence of GelE following culture in BHI (see ΔGS/pSprE) lacks proteolytic activity against this substrate. Arrows at the right indicate pro-SprE (closed) and processed SprE (open). Migration of several molecular weight standards are shown on the left.

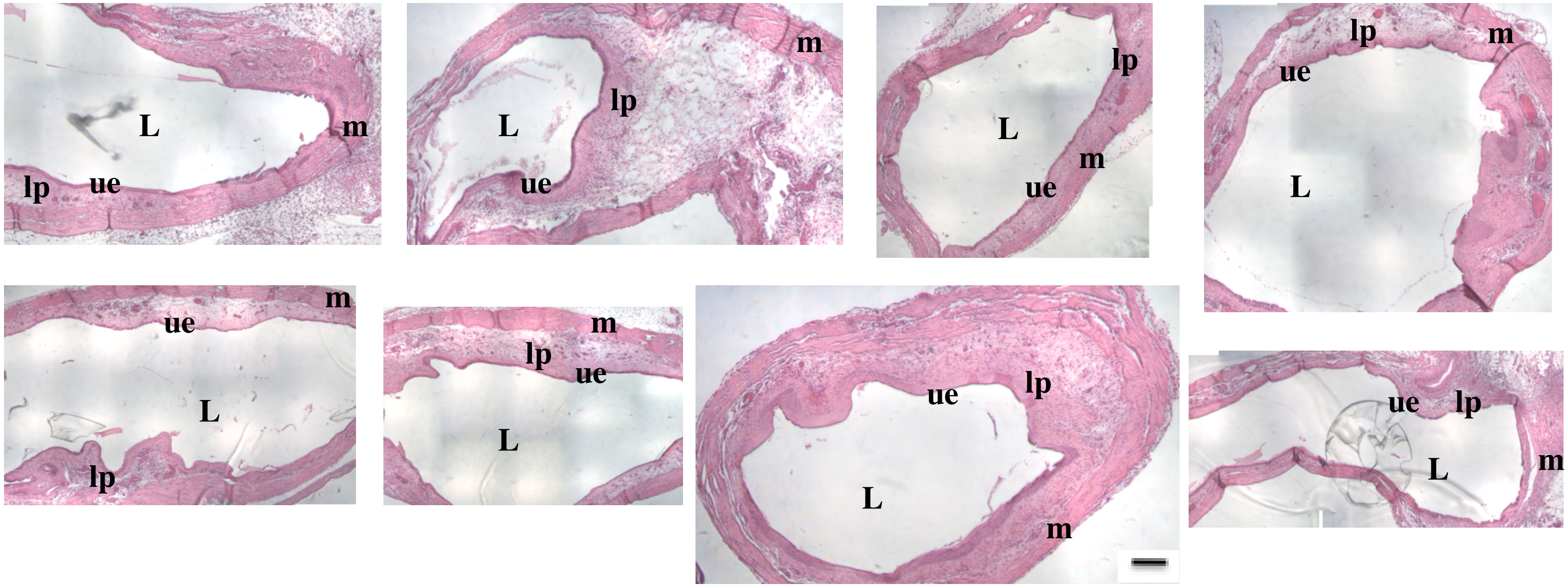

Figure S3. E64 treatment inhibits bladder inflammation. Bladders from mice (N=8) were treated with E64, implanted with catheters and infected with *E. faecalis* OG1RF according to the schedule described in Fig. 5C. Bladders were harvested, fixed, paraffin embedded, sectioned and stained with Hematoxylin and Eosin (H&E). A representative midsagittal section of each bladder is shown. For reference, the features of the bladder at the top left are labeled: **L**, lumen; **lp**, lamina propria; **ue**, urothelium; **m**, muscularis. Scale bar: 200μm

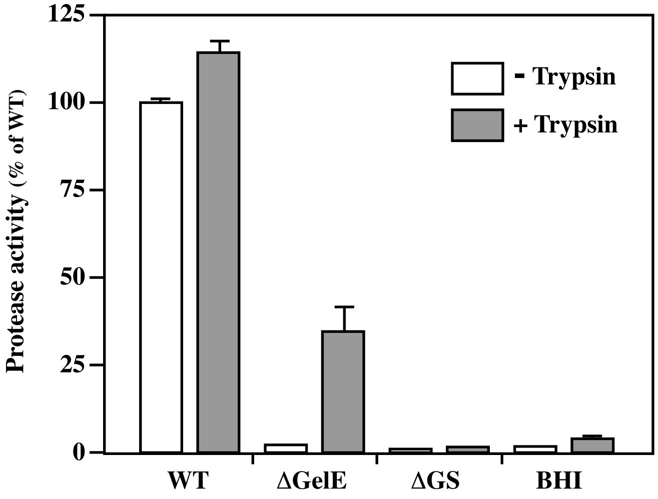

Figure S4. Trypsin can activate SprE *in vitro*. The indicated strains were inoculated in BHI media in the absence or presence of trypsin (1:500,000 final dilution of a 25 g/L stock solution) and cultured overnight. Protease activity of cell-free supernatants was then determined as described above and compared to uninoculated media with or without trypsin (BHI). This revealed that trypsin activated protease activity in the strain that produces SprE, but GelE (ΔGelE), but not in a strain lacking both SprE and GelE (ΔGS). Data are presented relative to WT grown in BHI medium without added trypsin. Shown is the mean ± SEM from a minimum of 3 independent experiments.

Table S1. Bacteria used in this study

Bacterial strain (alternative name):	Relevant Genotype:	Comment^:	source:
WT (OG1RF, WX1)	GelE+SprE+	express both GelE and SprE	(1)
ΔSprE (WX3)	Δ*sprE37-861*	Have GelE	This study
ΔGelE (WX5)*	Δ*gelE*	Have SprE	(2)
ΔGelE-SprE (WX7)	Δ*gelE-sprE24-2413*	No protease activity	This study
ΔGelE-SprE/pGelE-SprE (WX53)	Δ*gelE-sprE/pgelE-sprE-HA*	KanR, CamR, GelE-SprE-HA expressed on multicopy plasmid	This study
ΔGelE/pGelE (WX65)	Δ*gelE/pgelE-HA*	KanR, CamR, GelE-HA expressed on multicopy plasmid	This study
ΔSprE/pSprE (WX71)	Δ*sprE/psprE-HA*	KanR, CamR, SprE-HA expressed on multicopy plasmid	This study
ΔGelE-SprE/pGelE (WX77)	Δ*gelE-sprE/pgelE-HA*	KanR, CamR, GelE-HA expressed on multicopy plasmid	This study
ΔGelE-SprE/pSprE (WX83)	Δ*gelE-sprE/psprE-HA*	KanR, CamR, SprE-HA expressed on multicopy plasmid	This study
ΔFsrBD (WX103)	Δ*fsrBD64-681*	No protease activity	This study

*ΔGelE is called VT20 in (2).
^antibiotic resistances are abbreviated as follows: Kanamycin (KanR), Chloramphenicol (CamR)
GelE-Loci: OG1RF_11526
SprE-Loci: OG1RF_11525

REFERENCES

(1) Murray BE, Singh KV, Ross RP, Heath JD, Dunny GM, Weinstock GM. 1993. Generation of restriction map of *Enterococcus faecalis* OG1 and investigation of growth requirements and regions encoding biosynthetic function. *J Bacteriol*. 175:5216-23.

(2) Thomas VC, Hiromasa Y, Harms N, Thurlow L, Tomich J, Hancock LE. 2009. A fratricidal mechanism is responsible for eDNA release and contributes to biofilm development of *Enterococcus faecalis*. *Mol Microbiol* 72:1022-36.

Table S2. Primers used in this study

Primer Number	Name	Sequence	Description
ZCH#22	5' SprE upstream	CGAGGTCGACGGTATCGATAAGCTTGAAAAACAGGTATTCGAAATTTACAAAC	SprE pJRS233 Construct 5' Upstream
ZCH#23	3' SprE upstream	GGTTCTTTACGCTGCTGGCACAGCGGGAACTTTTTCATAAGATCATGCC	SprE pJRS233 Construct 3' Upstream
ZCH#24	5' SprE downstream	CCGCTGTGCCAGCAGCGTAAAGAACC	SprE pJRS233 Construct 5' Downstream
ZCH#25	3' SprE downstream	CGGCCGCTCTAGAACTAGTGGATCCGAAGATTGTTTTATTTTCGTTATAGACG	SprE pJRS233 Construct 3' Downstream
ZCH#207	5' GelE-SprE upstream	GGTCGACGGTATCGATAAGCTTGACGATGGTAAAGATGCTTTTTTATC	SprE GelE pJRS233 knock out vector 1
ZCH#208	3' GelE-SprE upstream	GATGAAGGGAAATAAAATTTTATACTCCGCTGTGCCAGCAG	SprE GelE pJRS233 knock out vector 2
ZCH#209	5' GelE-SprE downstream	CTGCTGGCACAGCGGAGTATAAAATTTTATTTCCCTTCATC	SprE GelE pJRS233 knock out vector 3
ZCH#210	3' GelE-SprE downstream	CCGCTCTAGAACTAGTGGATCCCACAAGAGTTTGTGATAACAAATAGTG	SprE GelE pJRS233 knock out vector 4
WX#1	5'GelE-pABG5	GACGCGGAATTCaatacttttgttgggaaaag	EcoRI site for clone into pABG5, RBS included
WX#2	3'GelE-HA-pABG5	AAAACTGCAGTCAAGCATAATCTGGAACATCATATGGATAttcattgaccagaacagatt	PstI site for clone into pABG5, HA tag after stop codon removed
WX#3	5'SprE-pABG5	GACGCGGAATTCaaaaaattccccaattaaat	EcoRI site for clone into pABG5, RBS included
WX#4	3'SprE-HA-pABG5	AAAACTGCAGTCAAGCATAATCTGGAACATCATATGGATAcgctgctggcacagcggata	PstI site for clone into pABG5, HA tag after stop codon removed
WX#9	5'GelE internal	acaatgctttttgggatgga	sequencing
WX#10	3'SprE internal	gaatgactttcggcaggaac	sequencing
WX#11	5' OJOY3S	ggggtccctttccttgatag	pABG5 sequencing
WX#12	5'GelE internal II	tgttgcttctgataatccag	sequencing
WX#13	3'SprE internal II	acatgggtatctgaagattc	sequencing
WX#14	3' pABG5 seq	atcaatatactcagtcactg	sequencing
WX#19	5' GelE#1-RT-PCR	CGCCATCACTAGCGACATTA	RT-PCR
WX#20	3' GelE#1-RT-PCR	CTTTTCCATCCCAAAAAGCA	RT-PCR
WX#21	5' GelE#2-RT-PCR	TTGATGCAATCGGTGAAGAA	RT-PCR
WX#22	3' GelE#2-RT-PCR	AAACCAATGATACGGGGTCA	RT-PCR
WX#23	5' SprE#1-RT-PCR	GCGTCAATCGGAAGAATCAT	RT-PCR
WX#24	3' SprE#1-RT-PCR	CATCTTTGGCATTCGGATTT	RT-PCR
WX#25	5' SprE#2-RT-PCR	TGTAGCTTTTTCCCCGAATG	RT-PCR
WX#26	3' SprE#2-RT-PCR	GCGTGTGGTTTTTCTCACCT	RT-PCR
WX#27	5' 16S rRNA#1-RT-PCR	AAGCAACGCGAAGAACCTTA	RT-PCR
WX#28	3' 16S rRNA#1-RT-PCR	GTCTCGCTAGAGTGCCCAAC	RT-PCR
WX#29	5' 16S rRNA#2-RT-PCR	CCATGTGTAGCGGTGAAATG	RT-PCR
WX#30	3' 16S rRNA#2-RT-PCR	CGGAAACCCTCCAACACTTA	RT-PCR
WX#31	5' 16S rRNA#3-RT-PCR	AGCGTTGTCCGGATTTATTG	RT-PCR
WX#32	3' 16S rRNA#3-RT-PCR	CATTTCACCGCTACACATGG	RT-PCR
WX#33	5' 16S rRNA#4-RT-PCR	ACCCATCAGAGGGGGATAAC	RT-PCR
WX#34	3' 16S rRNA#4-RT-PCR	CCGAAGATTCCCTACTGCTG	RT-PCR
WX#35	F-M13-fsrB ups A	tgtaaaacgacggccagtCTGCATCCAGATTTGATTAGAG	FsrB Phusion SOE-M13F KO1 F
WX#36	R-fsrB-dms A'-ups B	CAGTTTGTCCCATCCATTGttttcctgattgatcttcctg	FsrB Phusion SOE KO2 R
WX#37	F-fsrB-ups B-dms A'	caggaagatcaatcaggaaaaCAATGGATGGGACAAACTG	FsrB Phusion SOE KO3 F
WX#38	R-M13R- fsrB dms B'	CACAcaggaaacagctatgacCCATAAGCAATGTAAAAACGG	FsrB Phusion SOE-M13R KO4 R
WX#39	F fsrB sequencing	CATCGTGTGTCAATGGTTG	FsrB Verify Deletion F
WX#40	R fsrB sequencing	CAATCCATAATAATTTATTTTG	FsrB Verify Deletion R
WX#41	M13F (-40) colony PCR	gttttcccagtcacgac	pGCP213 colony PCR to confirm upper +downstream ligates
WX#42	M13R (+107) colony PCR	ggtgcctaatgagtgagctaac	pGCP213 colony PCR to confirm upper +downstream ligates
WX#43	5' fsrB#1-RT-PCR	TTTTATTGGTATGCGCCACA	RT-PCR
WX#44	3' fsrB#1-RT-PCR	CATCAGACCTTGGATGACGA	RT-PCR
WX#45	5' fsrB#2-RT-PCR	ATTTGGGACGTTAAGCGAAA	RT-PCR
WX#46	3' fsrB#2-RT-PCR	TGTGGCGCATACCAATAAAA	RT-PCR
WX#47	5' fsrA#1-RT-PCR	CGATCATCGTGTGTCAATGG	RT-PCR
WX#48	3' fsrA#1-RT-PCR	TGGTACTTCCGTTCCGTCTC	RT-PCR
WX#49	5' fsrA#2-RT-PCR	GCAAAAGCGTTAGAAGCATTG	RT-PCR
WX#50	3' fsrA#2-RT-PCR	TCCTGCCTGGATATGATTGTT	RT-PCR

Table S3. Effect of protease inhibitors on urine protease activity

Inhibitor	Protease inhibited	Inhibition of Fg cleavage[1]	Minimum concentration[2]
Leupeptin	serine/cysteine/ threonine	+	100-250 μg/ml
Pefabloc SC	serine	+	0.04-0.2 mg/ml
E64	cysteine	−	>50 μg/ml
EDTA	metaloprotease	−	>10 mM
Trypsin inhibitor	Trypsin/serine	+	40-200 μg/ml
Bestatin	amino-peptidase	−	>100 μg/ml
Aproptinin	serine	+	4-20 μg/ml
Pepstatin	aspartyl	−	>8 μg/ml
3,4-DCI	serine	+	100-500 μg/ml
Anti-thrombin	thrombin	−	>18 μg/ml

[1]Fg was added to normal human urine (1mg/ml final concentration) in the presence of the indicated inhibitor. Following 48hrs of incubation, Fg clevage was assessed by SDS-PAGE. A "+" indicates that no cleavage of Fg was observed. A "-" indicates that a cleavage pattern identical to untreated urine was observed. Experiments were repeated using urines from 2 different donors.

[2]The minimum concentration of inhibitor required to prevent any apparent Fg cleavage. In the cases where inhibition was not observed, the highest tested concentration is listed.

Table S4. Protease and protease inhibitors identified on uninfected and infected catheters.

Identified protein	Accession number	MW	Abundance[1]		
			Uninfected	Infected	Ratio[2]
**Proteases altered by infection**					
Trypsin precursor	gil|36429	24 kDa	0.10%	N.D.	≤ 0.10
**Inhibitors not altered by infection**					
Alpha-1-antitrypsin 1-4 precursor	gil6678085	46 kDa	0.36%	0.37%	1.03
Serine peptidase inibitor, clade A, member 1C	gil14602605 (+8)	46 kDa	0.31%	0.33%	1.06
Serine protease inhibitor A3K precursor	gil48747546	47 kDa	0.36%	0.42%	1.17
Antithrombin-III precursor	gil8252782	52 kDa	0.15%	0.09%	0.60
Cluster of alpha-1-antitrypsin 1-4 precursor	gil6678085 [11]	46 kDa	0.46%	0.65%	1.40
**Inhibitors altered by infection**					
Alpha-1 antitrypsin precursor	gil309079	46 kDa	N.D.	0.37%	≥ 38
Serine protease inhibitor A3N precursor	gil30503301 (+4)	47 kDa	N.D.	0.28%	≥ 28

[1]Proteins were extracted from catheters recovered following 24hrs of mock (uninfected) or infection by *E. faecalis* OG1RF and identified by Mass spectrometry as described in the Supplemental Methods. Shown is the relative abundance determined as a percentage of identified peptides corresponding to the indicated protein relative to the total number of identified peptides in the sample. N.D., no matching peptides detected.

[2]Ratio of infected vs. uninfected. Samples were no peptides were detected were assigned a value of 0.01% to determine a minimum estimate of the ratio.
